# An efficient in vitro regeneration system from different wild apple (*Malus sieversii*) explants

**DOI:** 10.1186/s13007-020-00599-0

**Published:** 2020-04-21

**Authors:** Y. Zhang, T. A. Bozorov, D. X. Li, P. Zhou, X. J. Wen, Y. Ding, D. Y. Zhang

**Affiliations:** 1 CAS Key Laboratory of Biogeography and Bioresource in Arid Land, Xinjiang Institute of Ecology and Geography, Urumqi, 80031 China; 2 Institute of Genetics and Plants Experimental Biology, Uzbek Academy of Sciences, Yukor-Yuz, Kibray Districts, 111226 Tashkent Region, Uzbekistan; 3 University of Chinese Academy of Sciences, Beijing, 100049 China; 4 Turpan Eremophytes Botanical Garden, Chinese Academy of Sciences, Turpan, 838008 China

**Keywords:** Dark treatment, Hormone concentration, Leaf and shoot explants, Leaf side orientation, *Malus sieversii*, Tissue culture

## Abstract

**Background:**

Wild apple, *Malus sieversii*, is an endangered species and a valuable genetic resource that requires a variety of conservation techniques. This study aimed to investigate the influence of different concentrations of hormones on wild apple regeneration from leaf and stem explants to establish an optimal regeneration system.

**Results:**

Leaves and stems derived from seedlings were cultured on several media supplemented with various concentrations of thidiazuron (TDZ) or 6-benzylaminopurine (BA) in different combinations with 1-naphthaleneacetic acid (NAA). The results showed that the most efficient shoot formation media (35% and 90%) were MS medium containing 4.0 mg L^−1^ TDZ and 1.0 mg L^−1^ NAA for leaf explants and MS medium containing 1.0 mg L^−1^ BA without NAA for stem explant. MS medium supplemented with 0.4 mg L^−1^ BA and 0.1 mg L^−1^ NAA (for shoot multiplication) and 1/2 MS + 0.1 mg L^−1^ NAA + 1.5% sucrose (for rooting) were effective media. Shoot regeneration from leaf explants was the most effective when the explants were placed abaxial side down onto the medium and were subjected to a pre-treatment of 3 weeks in darkness.

**Conclusions:**

An optimized regeneration system for *M. sieversii* that allowed regeneration within 2–3 months developed. The protocol developed herein can be used in large-scale clonal propagation for the conservation of wild apple, *M. sieversii*.

## Background

The forests of the Tianshan Mountains have a rich diversity of fruit trees. The Chinese part of Tianshan Mountains is estimated to be 38% wild fruit forest, whereas the Central Asian portion is estimated to be 62% wild fruit forest. These forests play an important ecological role in regulating climate, conserving water resources and preserving. Wild apple, *Malus sieversii* (Ledeb.) Roem., is native to the mountains of Central Asia and is widely distributed in the wild fruit forests of Tianshan Mountains. Wild apple forests are estimated to account for 92% of the fruit forests of the Chinese Tianshan Mountains, whereas wild apple forests make up approximately 78% of the wild apple forests in the Central Asian Tianshan Mountains [1].

Evolutionarily, the wild apple population has evolved cold-tolerant and disease-resistant varieties with diverse fruit coloration, shapes, and flavours [1]. Due to its abiotic stress tolerance, *M. sieversii* has been given wide attention in horticulture and is mainly used as rootstock in Northwest China and other provinces [2]. Several genetic studies have shown that the wild apple *M. sieversii* is the distant ancestor species of all cultivated domesticated apple species [3–5].

Unfortunately, in the last two decades, wild apple trees in Tianshan forests have been heavily damaged because of biotic stresses that have resulted in whole forest mortality. There are a number of reasons for wild apple mortality: the invasive insect *Agrilus mali*, fungal pathogens and human interference [6–9]. This tree has been listed as an endangered second-class protected plant in China. Therefore, this precious germplasm urgently needs effective protection [10], and it is necessary to create new stress-resistant germplasms. Therefore, the *ex* and in situ conservation of wild apple populations is globally important [11].

Currently there is the need to prevent the spread of invasive insects and generate apple trees that resistant to multiple stresses; however, traditional pruning and the use of pesticides are not effective for tree conservation. Alternatively, effective biotechnological methods, mainly in vitro clonal propagation methods, can be applied. These methods have been widely used in apple and pear horticulture. Additionally, these methods can be used for the propagation of stress-tolerant somaclonal lines. Several in vitro micropropagation protocols have been established for apple by several research groups [12–16]. There has been research on micropropagation or tissue culture methods for *M. sieversii*. The current study was focused on the establishment of an efficient in vitro micropropagation protocol for *M. sieversii* using a shoot regeneration system. The effects of the regeneration medium, explant type, leaf side orientation, and dark treatment on regeneration efficiency were studied. Different media for multiplication, shoot proliferation and rooting were also evaluated.

## Results

### Influence of different auxin and cytokinin concentrations on callus initiation in different explants

Leaf and stem explants from 30-day-old in vitro propagated shoots were used for callus induction. Both explants initiated callus formation on shoot induction medium (SIM) media containing various concentrations of cytokinin and auxin. To identify the most efficient medium composition for plant regeneration from leaf and stem explants, different hormone ratios of concentrations were examined with an orthogonal method (Table 1). Overall, explants can produce calli on SIM media depending on hormone concentrations and combinations (Additional file 1).

**Table 1:** Callus induction media used for rapid shoot induction in wild apple

Media	Concentration mg L^−1^		M	Concentration mg L^−1^	
	BA	NAA		TDZ	NAA
M1	1.0	0.0	M26	1.0	0.0
M2		0.5	M27		0.5
M3		1.0	M28		1.0
M4		1.5	M29		1.5
M5		2.0	M30		2.0
M6	2.0	0.0	M31	2.0	0.0
M7		0.5	M32		0.5
M8		1.0	M33		1.0
M9		1.5	M34		1.5
M10		2.0	M35		2.0
M11	3.0	0.0	M36	3.0	0.0
M12		0.5	M37		0.5
M13		1.0	M38		1.0
M14		1.5	M39		1.5
M15		2.0	M40		2.0
M16	4.0	0.0	M41	4.0	0.0
M17		0.5	M42		0.5
M18		1.0	M43		1.0
M19		1.5	M44		1.5
M20		2.0	M45		2.0
M21	5.0	0.0	M46	5.0	0.0
M22		0.5	M47		0.5
M23		1.0	M48		1.0
M24		1.5	M49		1.5
M25		2.0	M50		2.0

**Fig. 1:**
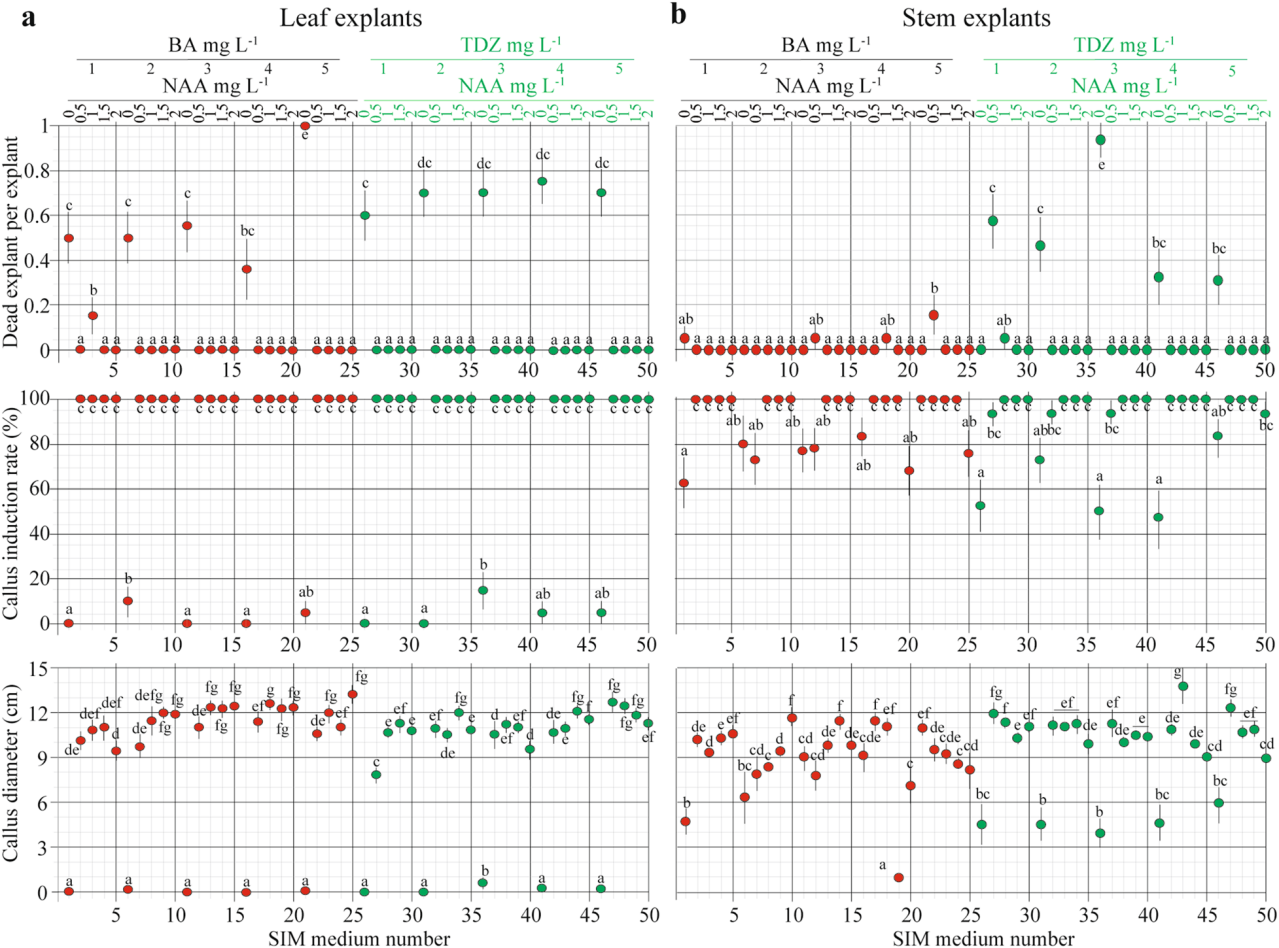
Effect of various SIMs on callus induction from leaf and stem explants. Calluses induced from leaf explants **a** and stem explants **b** after 4 weeks of cultivation. The values shown are the mean (± SE) of 20 explants. Different letters indicate significant differences among temperatures for each day as determined by Duncan’s multiple range test followed by one-way ANOVA at the p < 0.05 significance level. For the SIM number, see Table 1

Leaf explants dead on media supplemented with either BA or TDZ without NAA, and explant death ranged from 0.35 to 1 per explant (Fig. 1a). On these SIM, calli were not efficiently induced (15% induction), and callus diameter did not increase. Only 5% of explants produced calli on SIM supplemented with either TDZ or BA after 30 days of culture, and duration of this treatment was negatively correlated with the number of dead explants (Fig. 1a). A high concentration of BA (5 mg L^−1^, SIM 21) led to explant death. Most of the leaf explants showed callus formation after seven days, producing milky yellow-greenish callus from the edges of the explants on media containing both auxin and cytokinin. Callus formation was initiated continuously along the leaf vein over time and reached 100% after 15 days of post-culture on SIM supplemented with both cytokinin and auxin at different concentrations (Fig. 1a). Therefore, the addition of auxin was a pivotal regulator in the formation of calli from leaf explants. The callus diameter varied from 0.8 to 1.3 cm for both cytokinin and auxin (BA + NAA and TDZ + NAA), with an average of 11 cm (Fig. 1a).

**Fig. 2:**
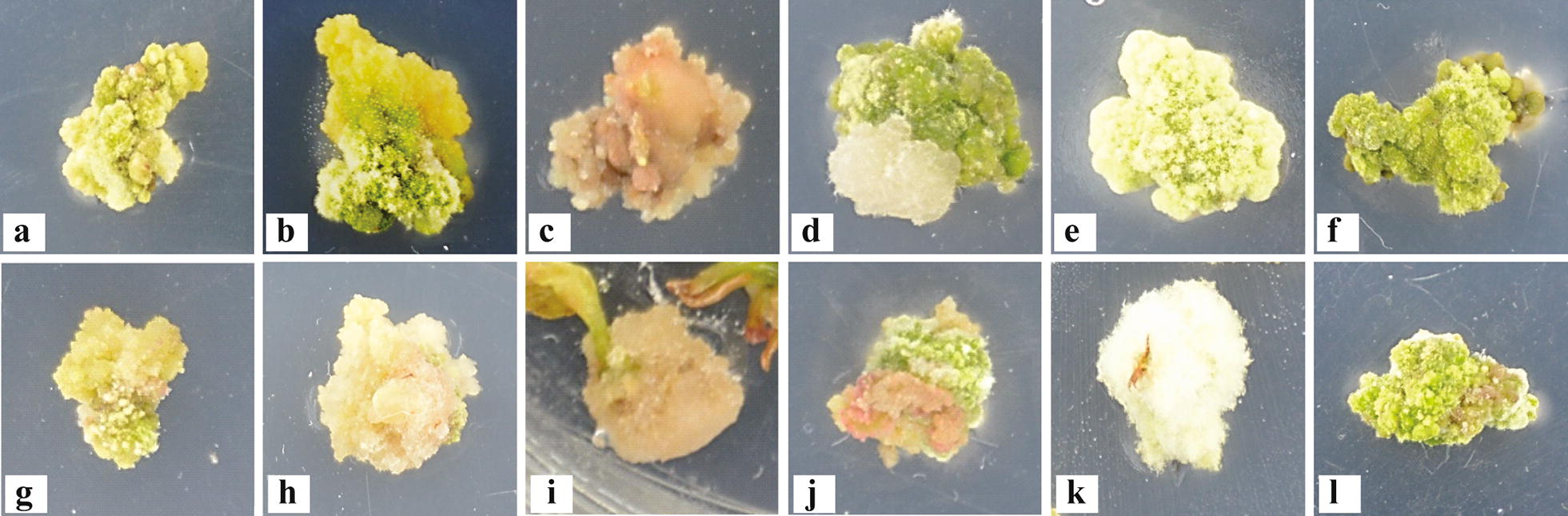
Colours and textures of calluses on SIM supplemented with various combinations of BA and TDZ. **a–f** Calluses obtained from 3-day-old leaf explants and **g–l** from stem explants. **a**, **g** Greenish-yellow, compact; **b** yellowish-green, friable; **c, i** brown, friable; **d** creamy white, friable; **e** white, short filamentous, compact; **f**, **l** green, compact; **h** creamy yellow, friable; **j** red, friable; **k** white, long filamentous, compact

Most of the calli induced from leaf explants were milky yellow, compact or friable (Fig. 2) during the initial incubation in the dark. After transferring calluses to a normal photoperiod (16 h day and 8 h night), the calli started growing larger in volume and becoming greener in colour. The type of cytokinin and the NAA concentration affected the characteristics of the callus. The callus was compact, and its coloration varied from light green to green in TDZ-supplemented media. With the addition of NAA at concentration of 0.5–1.5 mg L^−1^, the calli became white and filamentous. Unlike the TDZ-supplemented media, the BA-supplemented media produced tumour-like protrusions on the surface of the callus. At an NAA concentration of 1–2 mg L^−1^, the calli became white filamentous. In low concentration of NAA, cali were vitrified. After one or two months of culture, the calli demonstrated some nodular spots on the surface of the callus, and some of the calli were green with white filamentous covering (Fig. 2a–f). In some SIM, a yellowish green callus formed at the edge of compact green compact calli (Fig. 2b) to form an embryonic callus.

In contrast to leaf explants, stem explants showed good callus induction and a low death rate on SIM supplemented only with BA. Stem explants dead on TDZ-supplemented SIM, with death rate of 0.35 to 0.95 per explant, which was comparable to that of leaf explants on the same medium. However, compared to that of leaf explants, a 50 to 100% callus induction rate was observed on SIM supplemented only with TDZ or BA (Fig. 1a). This indicates that the stem can induce callus in the absence of auxin. Overall, callus induction from stem explants was faster than induction from leaf explants; callus induction from stem explants started after 10 days of culture on all SIM, and 100% of these explants survived 30-days of cultivation (Fig. 1b). The callus induction rate from the stem explants on BA + NAA media showed greater variation (70% and 100%) that that from leaf explants; greater variation was also observed in the diameter of calli induced from stem explants than from leaf explants.

The coloration of the calli on stem segments varied: most of the calli were greenish-yellow, and their texture was friable (Fig. 2 g^−l^), forming an embryonic callus. Brown calli were found on SIM without auxin, likely indicating the production of phenolics that led to callus death. The reduction of either BA or TDZ from 5 to 1 mg L^−1^ produced mainly green, compact calli.

### Influence of growth regulators on the regeneration rate

**Fig. 3:**
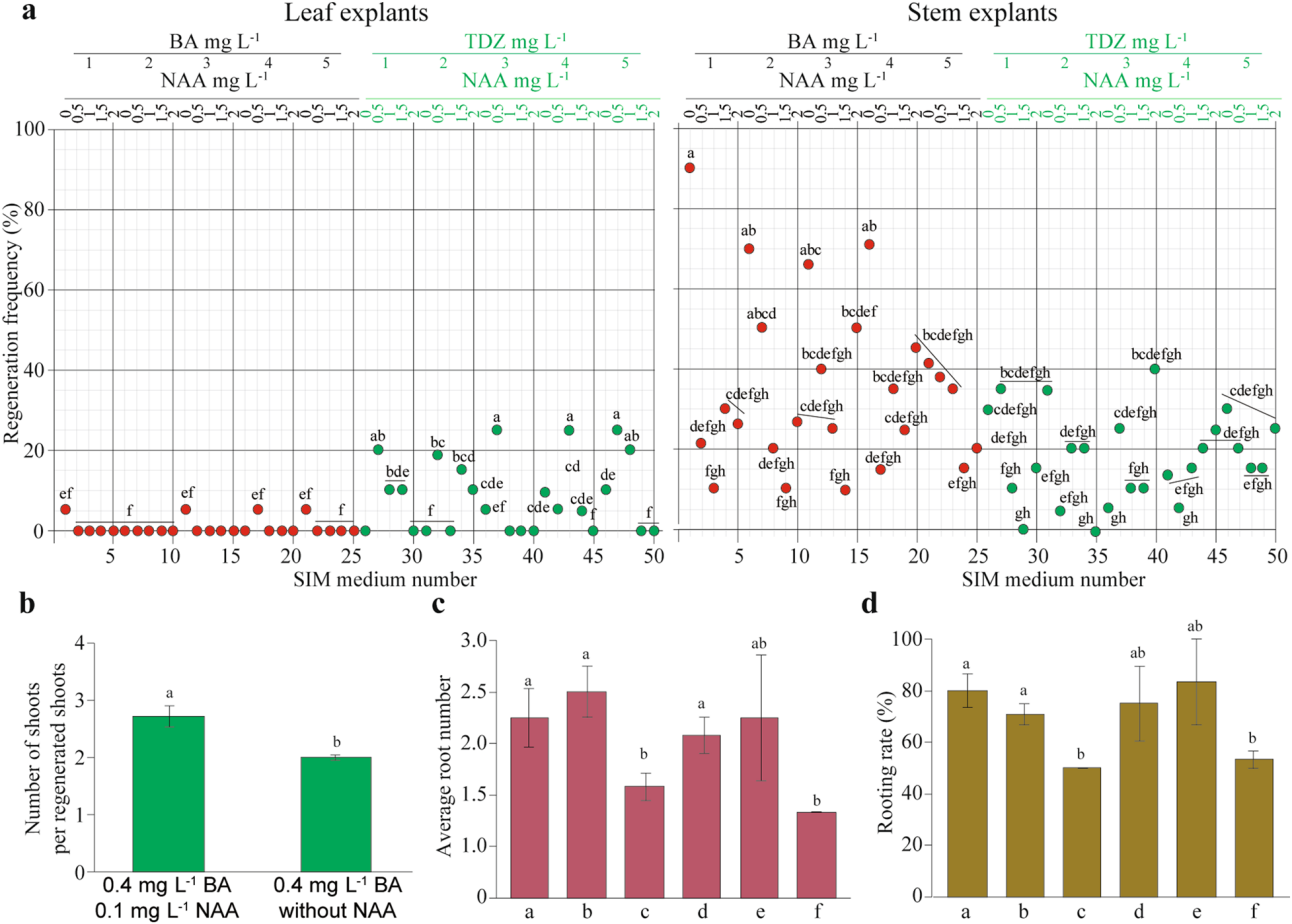
Influence of growth regulators on the regeneration rate from leaf and stem explants. **a** Regeneration rate from leaf and stem explants after sixty days of cultivation. **b** Elongation rate of shoots produced from leaf explants on two different media. **c** Average root number and **d** rooting rate produced from six different media supplemented with different concentrations of a carbon source. Lowercase letters on the x-axis indicate the hormone and sucrose concentrations described in Table 3. The values shown are the mean (± SE) of 20 explants. Different letters indicate significant differences among temperatures for each day as determined by Duncan’s multiple range test followed by one-way ANOVA at the p < 0.05 significance level

Growth regulators differently influenced somatic embryogenesis and axillary bud regeneration (Additional file 2). The regeneration rate from leaf and stem explants was dependent on the cytokinin type. The highest number of regenerated shoots per explant was observed in stem explants (Additional file 2). In leaf explants, BA did not strongly influence the regeneration rate (0.05–0.25 shoots per explant; Additional file 3). After 30 days of cultivation on SIM supplemented with 1–5 mg L^−1^ BA without NAA, the regeneration frequency was 5% of (Fig. 3a), but the shoots were the least developed of those in all treatments (Additional file 2). The addition of NAA at different concentrations into SIM with BA did not lead to shoot protection. In contrast to BA, TDZ influenced the regeneration rate of leaf explants. TDZ at concentrations of 1 and 2 mg L^−1^ was not able to induce regeneration, but when the concentration was increased from 3 to 5 mg L^−1^, the explants produced regenerated shoots at a rate of up to 10%. However, the addition of 2 mg L^−1^ NAA together with TDZ negatively affected shoot regeneration except with TDZ at a concentration of 2 mg L^−1^. Regeneration was observed when both growth regulators were added into SIM, and explants were able to produce regenerated shoots at a rate of 5 to 25%. In particular, a moderate concentration of 0.5–1 mg L^−1^ NAA with TDZ led to the production of regenerated shoots.

**Fig. 4:**
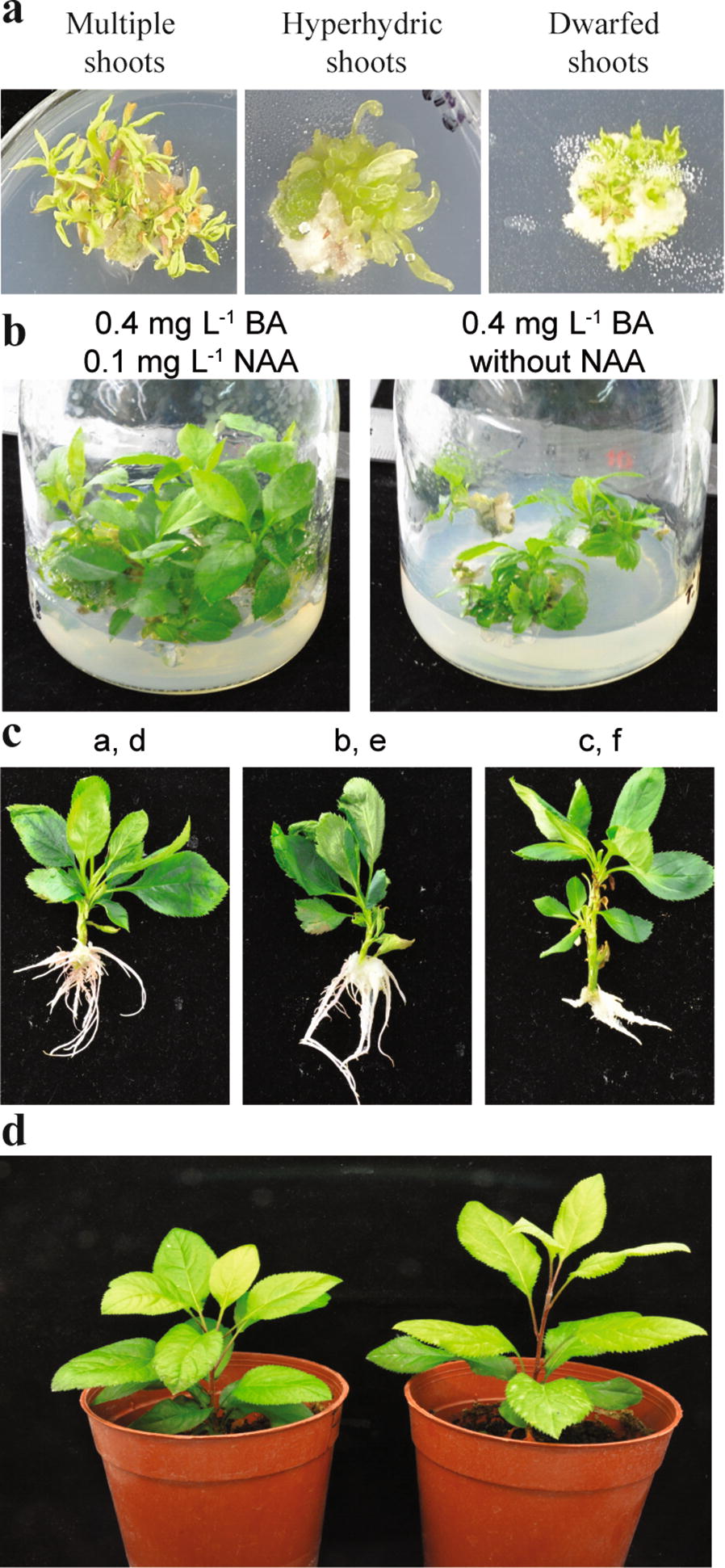
Effect of growth regulators on the shoot elongation and rooting. **a** Types of the regenerated shoots from SIM. **b** Photos depict elongation of the regenerated shoots in different SMM. **c** Rooting of the explants obtained from SRM. Letters indicate SRM in Table 3. **d** Transferred plantlets into potting medium

Stem segments could regenerate after 15–20 days of culture on SIM, but the majority of stem regeneration was observed after 60 days with maximum 0.85 shoots per the explant (Additional file 3). In contrast to leaf explants, stem explants produced shoots in media supplemented with BA. The highest shoot regeneration rate was achieved on SIM supplemented only with BA. With a decrease in the BA concentration without NAA, the regeneration rate increased from 55 to 90%, and multiple shoots developed from stem explants that grew simultaneously and normally. Regeneration occurred within 2 weeks of post-culture and likely regenerated from axillary buds. However, hyperhydric shoots developed in SIM supplemented only with BA at high concentrations (5 mg L^−1^) (Fig. 3a; Additional file 2). With the addition of various concentrations of NAA to SIM containing BA, the regeneration rate decreased from 55 to 10%. Hormone combinations with different concentrations influenced the regeneration rate. For example, the combination of BA with NAA demonstrated regeneration rates of up to 50% with 0.5 mg L^−1^ NAA, up to 35% with 1 mg L^−1^ NAA, up to 30% with 1.5 mg L^−1^ NAA, and up to 50% with 2 mg L^−1^ NAA. However, in SIM with high concentrations of BA (5 mg L^−1^) and NAA (2 mg L^−1^), the shoots were compact and poorly developed. Mainly, these regenerated shoots originated from developed calli. Moreover, NAA could have negative interaction with BA in axillary shoots regeneration. Brown calli were mostly observed in SIM supplemented with 1 and 1.5 mg L^−1^ NAA, which had a negative impact on the regeneration rate. SIM containing only TDZ at different concentrations showed a lower regeneration rate (5 to 35%) and increased hyperhydric shoots comparing SIM containing only BA (Fig. 4a). Overall, the regeneration rate in TDZ-supplemented media was lower than that in media containing BA with the same NAA concentration (Fig. 3a; Additional file 2). The addition of various concentrations of NAA to SIM containing TDZ reduced the regeneration rate and worsened shoot development. The shoots on these SIMs were dense, dwarfed, and hyperhydric and had difficulty to elongating (Fig. 4a; Additional file 2). In summary, SIM containing 1 mg L^−1^ BA without NAA was chosen as the best medium for regeneration from stem segments, with up to 90% regeneration rate.

### Effect of growth regulators on dwarf shoot multiplication and rooting

Optimizing the growth of regenerated dwarf shoots obtained from SIM supplemented with either TDZ or a high concentration of BA was essential for transferring the shoots onto shoot multiplication medium (SMM) for normal development. For this purpose, we examined two different SMM containing BA (0.4 mg L^−1^) with or without NAA (0.4 mg L^−1^) (Table 2). The results showed that dwarf shoots multiplied significantly multiplied more on SMM containing both BA and NAA (3.36 ± 0.21) than on SMM without NAA (2.5 ± 0.13) (Figs. 3b, 4b). Additionally, regenerated shoots in SMM supplemented with BAA and NAA elongated up to 5 cm; those SMM without NAA elongated to 3 cm.

**Table 2:** Hormone composition of SMM used in this study

BA concentration (mg L^−1^)	NAA concentration (mg L^−1^)
0.4	0.1
0.4	–

To obtain complete plantlets, six different SRM rooting media were designed. Additionally, different concentrations of sucrose (15 and 30 g L^−1^) were tested as carbon sources (Table 3). Rooting from regenerated shoots demonstrated that sucrose did not have much influence after 15 days of culture. The increase in NAA concentration significantly influenced the number of roots (Figs. 3c, 4c). Similarly, the rooting rate demonstrated the same pattern as the number of roots (Fig. 3d). Plantlets with well-developed shoots and roots were transferred to a soil mixture (Fig. 4d). Taken together, SRM with sucrose (15 or 30 g L^−1^) supplemented with 0.1 or 0.5 mg L^−1^ NAA was an effective protocol for rooting. The survival rate of plantlets transferred into pots was 83-100%.

**Table 3:** Auxin and sucrose combinations used in SRM

#	NAA concentration (mg L^−1^)	Sucrose concentration (g L^−1^)
a	0.1	15
b	0.5	15
c	1	15
d	0.1	30
e	0.5	30
f	1	30

### Effect of leaf side orientation and dark treatment on regeneration frequency

**Fig. 5:**
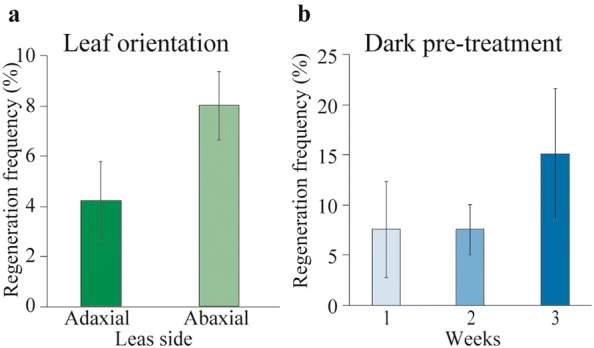
Effect of dark treatment on callus development in leaf explants orientated adaxially and abaxially on SIM M32. **a** Regeneration frequency of shoots produced from leaf callus when leaf explants were placed on the medium on their adaxial or abaxial side. **b** Regeneration frequency obtained from 30-day-old leaf explants pre-treated in the dark for 1, 2 and 3 weeks

To further to explore the influence of leaf side placement on the regeneration rate, we examined the effect of different leaf side orientations on the regeneration rate under a pre-treatment in the dark. The results showed that regeneration efficiency non-significantly increased and that the characteristics of the callus differed. The callus from the abaxial side of the leaf was light yellow and friable in texture, but the callus from the adaxial side of the leaf was white friable (Additional file 4). The inner mesophyll tissue of the adaxial-side lamina in contact with medium was prone to dryness and browning. The regeneration rate of the abaxial-side of the leaf explants was slightly higher than that of the adaxial-side of the leaf explants (Fig. 5a). In summary, leaf abaxial contact with the medium was effective for callus formation.

The effects of the duration of the dark treatment on the callus induction and regeneration rates were investigated. The results showed that 3 weeks of pre-treatment in darkness non-significantly increased the regeneration rate and increased the callus formation intensity compared to those after one or two weeks of pre-treatment in darkness (Fig. 5b).

## Discussion

**Fig. 6:**
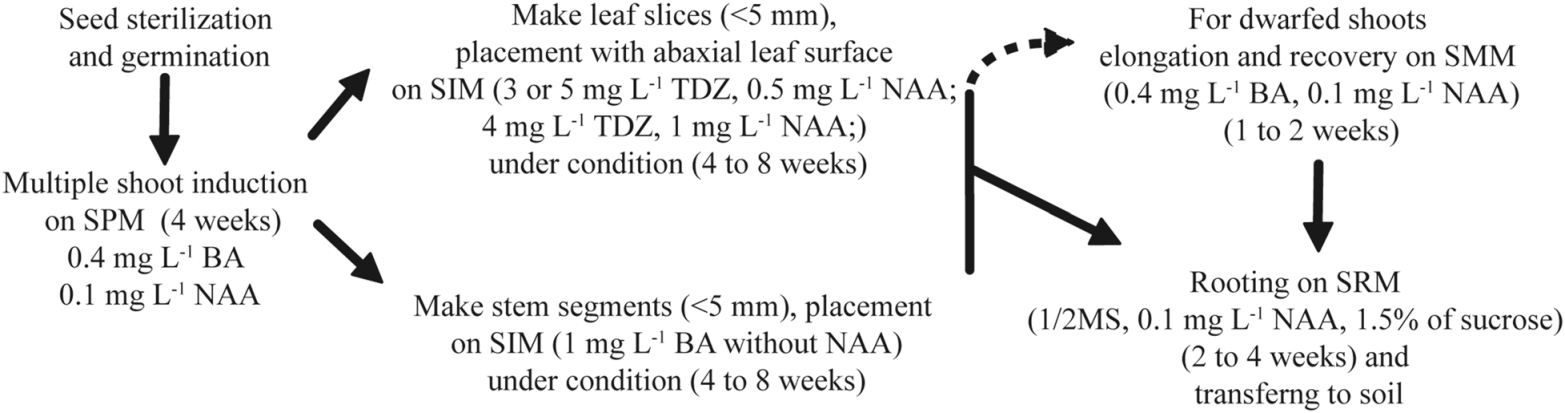
Schematic representation of wild apple *in vitro* rapid micropropagation

In this study, we optimized an effective rapid regeneration method from wild apple leaf and stem explants on media supplemented with various concentrations of hormones. A schematic representation of the procedure is depicted in Fig. 6. This method allows rapid propagation from either leaf or stem segments within a short time.

The cytokinin and auxin must be balanced during shoot regeneration from explants. The regeneration frequency from leaf explants is less than that from stem explants. In particular, high shoot regeneration from stem explants was achieved in media containing only cytokinin (BA) without auxin. It is likely that BA interacted directly with endogenous auxin in axillary buds to induce regeneration. Auxin produced in the stem, buds, and root tips promotes cell elongation. Auxin maintains apical dominance inhibiting the outgrowth of lateral buds. However, cutting off the apical meristem results a decline in apical dominance and triggers axillary bud development [17], which was observed in current study. It is likely that stem segment activates the production of endogenous auxin in lower amounts that co-regulate bud proliferation. This finding was supported by experiments in which poorly developed shoots were multiplied and developed more normally at low concentrations of auxin than in auxin free medium (Figs. 3b and 4b). This result could explain why shoot regeneration from stems occurred in medium supplemented only with BA [18, 19]. However, the addition of auxin into the media greatly decreased bud regeneration. This indicates that a high concentration of auxin inhibited direct shoot organogenesis but triggered somatic embryogenesis [20]. In contrast, leaves treated with cytokinin alone did not show regeneration or callus formation because there was no endogenous auxin. Shoot regeneration from leaves occurs through somatic embryogenesis, which normally requires both hormones.

Interestingly, media containing only TDZ was not effective for bud regeneration. This finding is consistent with work by Mohavedi et al. [21], who demonstrated the efficiency of BA compared to that of TDZ in stem regeneration. TDZ might not effectively regulate shoot formation and might interact negatively with endogenous auxin. TDZ is more effective than BA for leaf regeneration from explants, which was also demonstrated in apple by Li et al. [22]. TDZ induced abnormalities such as hyperhydric shoots, dwarfing, and shoot fasciation [23, 24]. However, the level of abnormality was dependent on the TDZ concentration as well as the apple genotype [25]. There is no direct relationship between shoot regeneration and callus size, there is a direct relationship between shoot regeneration and but callus features. Calluses with different characteristics can be formed under various combinations of hormone concentrations. The regeneration events are related to the re-differentiation ability of a small number of callus cells.

Light is one of the factors that affects the regeneration system. Our findings indicate that light suppressed callus genesis and adventitious shoot formation in the first step of culture, but a dark pre-treatment of the explants enhanced shoot formation. It has been reported that light treatment delays callus formation from thin cell layers in apple cultivars [26, 27]. However, the transverse thin cell layers of domestic apples used as explants under light showed higher shoot regeneration capacity than those under a dark treatment [28]. Additionally, adventitious shooting is species dependent [29]. Moreover, darkness negatively influences on leaf stomatal closure and leads to stomatal opening [30].

Apparently, leaf morphology plays an important role in in vitro growth responses. Placing the abaxial side of the leaf onto the medium enhances regeneration efficiency compared to placing the adaxial side onto the medium. Similar work conducted by Zhang et al. [16] showed that placing the abaxial side of the leaf touched onto the medium produced adventitious shoots from 96% of explants compared to 39% adventitious shoot production from adaxial-side the leaf explants. Several factors could influence regeneration: (1) the number of stomata on the adaxial side lower than that on the abaxial side, which could play a role in callus formation. Leaf stomatal opening on abaxial surfaces was unregulated in apple leaves cultured in vitro, and more than 95% of stomata were wide circular openings [31]. (2) The abaxial leaf side has more area than the adaxial side, which provides maximum nutrient absorption. This could be because greater number of fully open stomata on abaxial surface could absorb nutrients and hormones from the medium. (3) Stomatal guard cells could be totipotent. It has been successfully demonstrated that stomatal guard cells retain full totipotency in sugar beet [32]. The stomatal density is higher on the abaxial surface than on the adaxial surface [33]. This could also be the reason why the regeneration frequency from the abaxial side is higher than that from the adaxial side.

## Conclusions

Taken together, the current work demonstrates the optimization of a wild apple regeneration method using different concentrations and types of hormones as well as the influence of darkness and leaf side orientations on leaf and stem explants. A regeneration system for *M. sieversii* was established for the first time, and the shortest regeneration time achieved was 2.5 months. In summary, the optimal hormone concentrations for leaves explants were 4 mg L^−1^ TDZ and 1 mg L^−1^ NAA, and those for the stem explants were 1 mg L^−1^ BA without NAA. For the multiplication medium, the optimal hormone concentration was 0.4 mg L^−1^ BA and 0.1 mg L^−1^ NAA, and for the rooting medium, the optimal hormone concentration was 1/2 MS + 0.1 mg L^−1^ NAA + 1.5% sucrose.

## Methods

### Plant material

Wild apple (*M. sieversii*) seeds were collected from the Yili Botanical Research Station (43° 22  N 83° 34  W) located in the Ili-Kazakh district of the Xinjiang-Uyghur Autonomous Region, which is located the in Ili Valley of the Tianshan Mountains. The seeds were surface sterilized. Briefly, the fruit surface was treated with 75% ethanol, and the fruits were cut to remove seeds under a sterile laminar flow hood. The seeds coats were removed, and the seeds were washed three times with sterile water. The seeds were soaked in sterile water for 5 days and transferred onto Murashige–Skoog medium (QDRS BIOTEC, China) supplemented with 6 g L^−1^ agar. The seeds were germinated at room temperature and kept under a 16/8 h photoperiod at a light intensity of 25 μmol m^−2^ s^−1^ provided by fluorescent lamps. Seedlings with true leaves were used for micropropagation for further tissue culture experiments. The shoot tips were isolated and multiplied in vitro on shoot propagation medium (SPM) containing MS basal salts (0.4 mg L^−1^ BA, 0.1 mg L^−1^ NAA, 6 g L^−1^ agar, pH 5.8) [34] and vitamins and subcultured onto fresh medium every 8 weeks.

### Callus induction and regeneration

Thirty-day-old in vitro propagated shoots of *M. sieversii* seedlings with uniform unfolded leaves were used to initiate callus induction and shoot propagation. The petioles were removed, and the leaf laminas were cut transversely to the midrib into two parts. Leaf and stem explants < 5 mm in size were placed on a shoot induction medium (SIM) (Basal MS medium with vitamins, 30 g L^−1^ sucrose, 7 g L^−1^ agar, pH 5.8) containing various concentrations of thidiazuron (TDZ) or 6-benzylamino purine (BA) in different combinations with 1-naphthaleneacetic acid (NAA) (Table 1). Ten stem and leaf explants were placed in each Petri dish (94 mm in diameter) containing approx. 25 mL SIM for regeneration. In total, four plates were used for each SIM treatment. In the initial incubation, the explants were cultivated in the dark at 24 ± 1 °C for 3 weeks. Regenerated shoots were cultured at the same temperature with a photoperiod of 16/8 h day/night. Sub-cultivation was performed every 4 weeks and at least twice. The experiment was repeated twice. The explants survival rate, the callus and adventitious bud induction rates, and the callus and adventitious bud growth states were measured at four and eight weeks post-subculturing.

### Shoot multiplication

Regenerated poorly developed shoots from leaf explants were transferred into shoot multiplication medium (SMM). The SMM medium contained MS basal salts, vitamins, 0.4 mg L^−1^ BA with or without 0.1 mg L^−1^ NAA, and 3% (w/v) sucrose with an adjusted pH of 5.8 before sterilization (Table 2). The cultivation of shoots on SMM was performed in 300 mL disposable plastic vessels containing 100 mL medium. Five regenerated buds per container were placed in each vessel for propagation. After 30 days, when the shoots had attained 2 to 3 cm length, the multiplication rate was calculated based on the number of shoots derived from one bud cluster. There were thirty plants per treatment, and each treatment was repeated twice.

### Rooting

After the shoot multiplication medium, regenerated shoots of approximately 3 cm in length were individually placed on shoot rooting medium (SRM). The SRM medium contained 1/2 MS basal salts, and vitamins as well as different NAA and sucrose concentrations (Table 3). Altogether, six SRMs with hormone and sucrose combinations were tested. For each treatment, at least ten regenerated shoots with three repetitions were used. The root number and rooting rate were calculated at 30 days post-cultivation.

### Acclimatization and transplanting

Ten replicates of regenerated plantlets 5 cm in length with 5–10 roots were washed with water to remove any adhered agar and then transferred into a 1 L plastic pots. The potting medium was peat moss and vermiculite in a 3:1 (v/v) ratio. The pots with plantlets were covered with a plastic film to maintain a high moisture level. Initially, a sufficient amount of water was provided. The light intensity was half of that used during the tissue culture period. After 7 days, the film was halfway removed, and the light intensity was changed to normal lightning conditions. During this period, an amount of water sufficient for plant growth was sprayed under the film until the first new leaves of the plantlets completely expanded. After 2 weeks, the film was completely removed and the plantlets were cultivated under greenhouse conditions (25 °C day, 20 °C night with a 16/8 day/night photoperiod and 75% relative humidity) with occasional watering for 3 months.

### Dark treatment and leaf orientation experiment

One hundred leaf explants from 30-day-old in vitro propagated shoots were used to determine the efficiency of different leaf side orientations. For this purpose, explants with their adaxial or abaxial leaf side touching SIM (M32, Table 1) (basal MS medium with vitamins, 30 g L^−1^ sucrose, 7 g L^−1^ agar, pH 6.0 containing TDZ (4 mg L^−1^) with NAA (1 mg L^−1^) were cultivated in dark conditions for 1–3 weeks. Each treatment of 20 explants per Petri dish was repeated three times. The callus induction rate and adventitious bud regeneration rate were counted after culturing for 30 days.

### Statistical analysis

SPSS 23.0 statistics and analysis software was used to conduct Duncan’s test following one-way ANOVA.

## Supplementary information

**Additional file 1.** Callus induction in different SIM from 30-day-old leaf and stem explants.

**Additional file 2.** Adventitious shoots regenerated from 60-day-old leaf and stem explants from different SIM.

**Additional file 3.** The number of the regenerated shoots per explant.

**Additional file 4.** Induced callus from different leaf side orientations under a pre-treatment in the dark.

## Data Availability

The datasets used and/or analyzed during the current study are available from the corresponding authors on reasonable request.
