# Supplementary figures and images for: An efficient in vitro regeneration system from different wild apple (Malus sieversii) explants
Source: Plant Methods. 2020 Apr 21;16:56. doi: 10.1186/s13007-020-00599-0 (PMC7175559; doi:10.1186/s13007-020-00599-0)

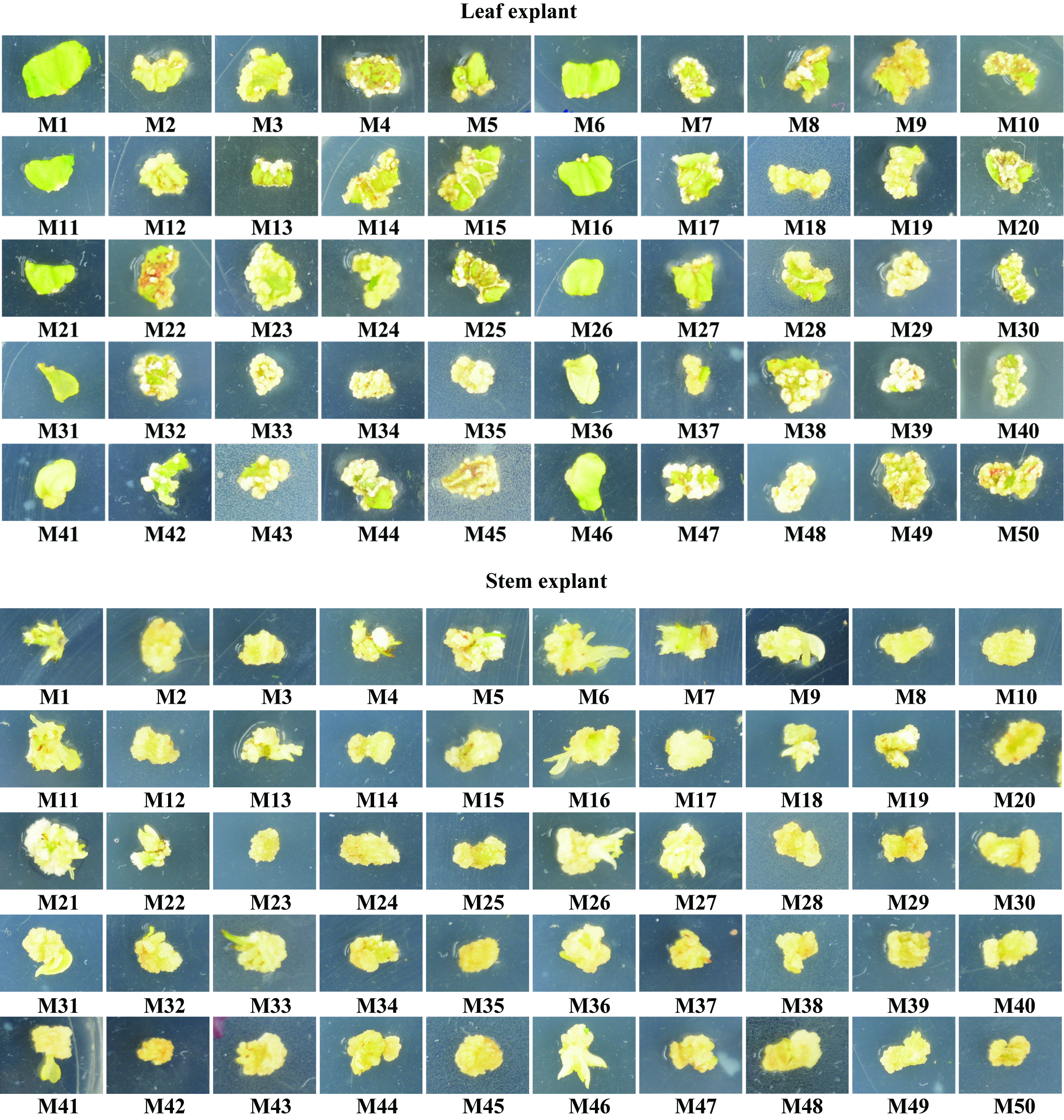

Supplement: Supplementary file 1 — Additional file 1. Callus induction in different SIM from 30-day-old leaf and stem explants. [file 13007_2020_599_MOESM1_ESM.tif]

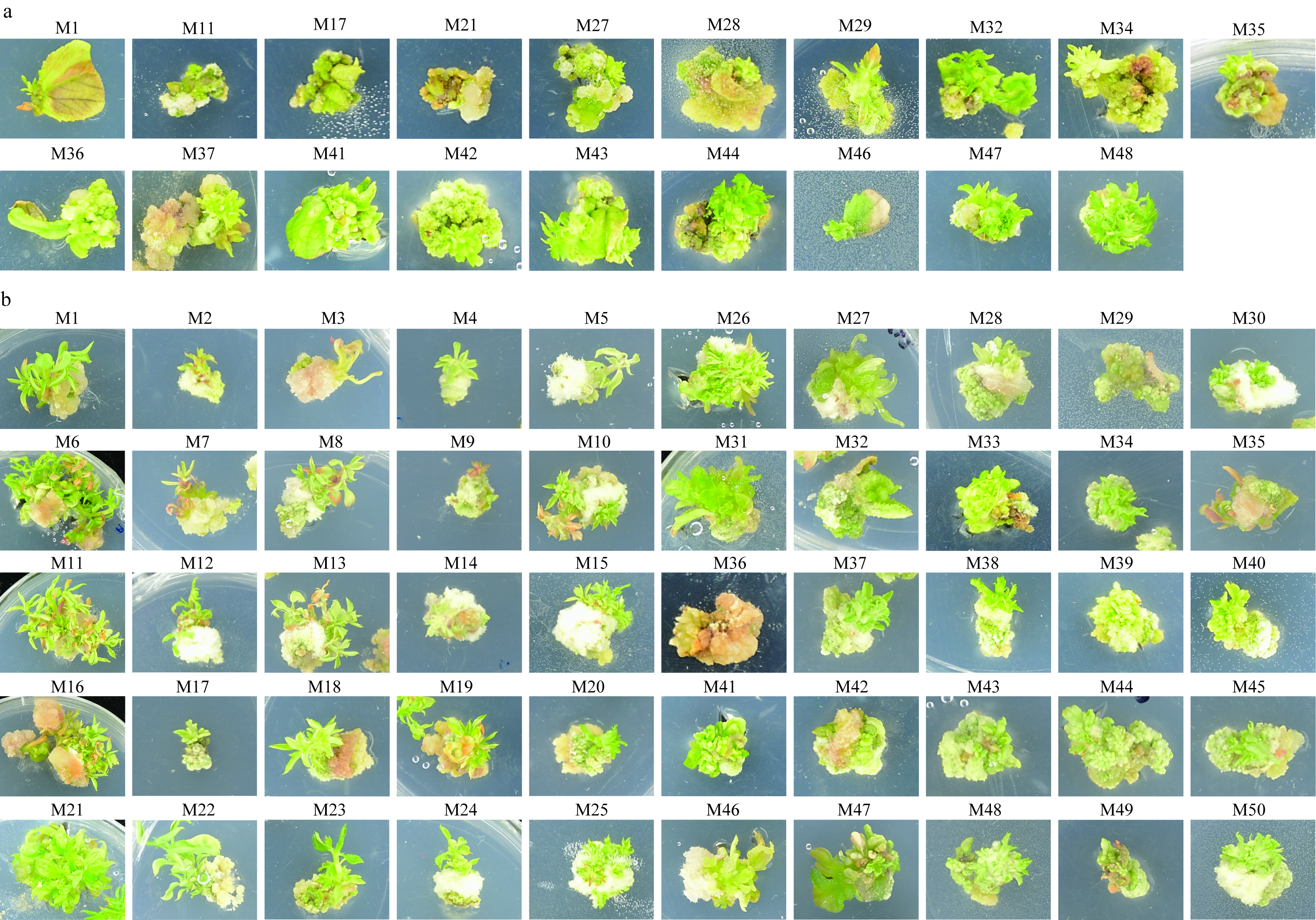

Supplement: Supplementary file 2 — Additional file 2. Adventitious shoots regenerated from 60-day-old leaf and stem explants from different SIM. [file 13007_2020_599_MOESM2_ESM.tif]

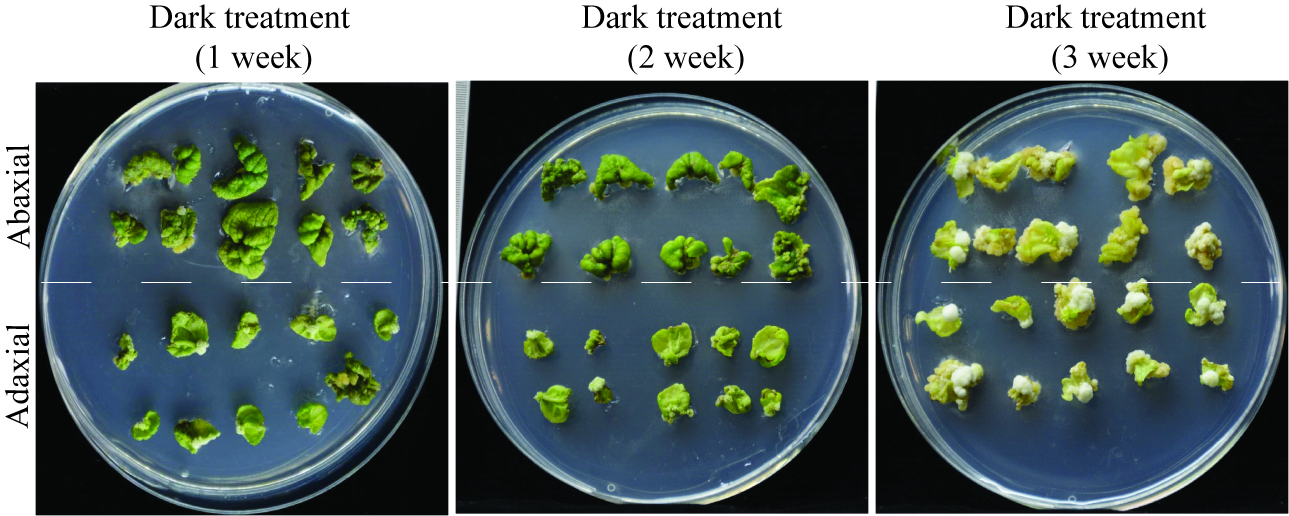

Supplement: Supplementary file 4 — Additional file 4. Induced callus from different leaf side orientations under a pre-treatment in the dark. [file 13007_2020_599_MOESM4_ESM.tif]
