# Supplementary material for: An efficient in vitro regeneration system from different wild apple (Malus sieversii) explants
Source: Plant Methods. 2020 Apr 21;16:56. doi: 10.1186/s13007-020-00599-0 (PMC7175559; doi:10.1186/s13007-020-00599-0)
Supplement: Supplementary file 3 — Additional file 3. The number of the regenerated shoots per explant. [file 13007_2020_599_MOESM3_ESM.docx]

| **#** | **SIM media** | **Stem** | | **Leaf** | | **#** | **SIM media** | **Stem** | | **Leaf** | |
| --- | --- | --- | --- | --- | --- | --- | --- | --- | --- | --- | --- |
|  |  | **regenerated shoots per explant** | **SE** | **regenerated shoots per explant** | **SE** |  |  | **regenerated shoots per explant** | **SE** | **regenerated shoots per explant** | **SE** |
| **1** | **6-BA 1 mg/l, NAA 0.0 mg/l** | 0.85 | 0.07 | 0.05 | 0.05 | **26** | **TDZ 1 mg/l, NAA 0 mg/l** | 0.30 | 0.06 | 0.20 | 0.00 |
| **2** | **6-BA 1 mg/l, NAA 0.5 mg/l** | 0.21 | 0.04 | 0.00 | 0.00 | **27** | **TDZ 1 mg/l, NAA 0.5 mg/l** | 0.35 | 0.09 | 0.10 | 0.08 |
| **3** | **6-BA 1 mg/l, NAA 1.0 mg/l** | 0.10 | 0.03 | 0.00 | 0.00 | **28** | **TDZ 1 mg/l, NAA 1.0 mg/l** | 0.10 | 0.03 | 0.10 | 0.06 |
| **4** | **6-BA 1 mg/l, NAA 1.5 mg/l** | 0.30 | 0.09 | 0.00 | 0.00 | **29** | **TDZ 1 mg/l, NAA 1.5 mg/l** | 0.00 | 0.00 | 0.00 | 0.06 |
| **5** | **6-BA 1 mg/l, NAA 2.0 mg/l** | 0.28 | 0.05 | 0.00 | 0.00 | **30** | **TDZ 1 mg/l, NAA 2.0 mg/l** | 0.15 | 0.04 | 0.00 | 0.00 |
| **6** | **6-BA 2 mg/l, NAA 0 mg/l** | 0.70 | 0.03 | 0.00 | 0.00 | **31** | **TDZ 2 mg/l, NAA 0 mg/l** | 0.35 | 0.04 | 0.20 | 0.00 |
| **7** | **6-BA 2 mg/l, NAA 0.5 mg/l** | 0.58 | 0.07 | 0.00 | 0.00 | **32** | **TDZ 2 mg/l, NAA 0.5 mg/l** | 0.05 | 0.02 | 0.15 | 0.12 |
| **8** | **6-BA 2 mg/l, NAA 1.0 mg/l** | 0.20 | 0.04 | 0.00 | 0.00 | **33** | **TDZ 2 mg/l, NAA 1.0 mg/l** | 0.20 | 0.04 | 0.10 | 0.00 |
| **9** | **6-BA 2 mg/l, NAA 1.5 mg/l** | 0.10 | 0.04 | 0.00 | 0.00 | **34** | **TDZ 2 mg/l, NAA 1.5 mg/l** | 0.20 | 0.06 | 0.05 | 0.05 |
| **10** | **6-BA 2 mg/l, NAA 2.0 mg/l** | 0.27 | 0.03 | 0.05 | 0.00 | **35** | **TDZ 2 mg/l, NAA 2.0 mg/l** | 0.00 | 0.00 | 0.25 | 0.06 |
| **11** | **6-BA 3 mg/l, NAA 0 mg/l** | 0.63 | 0.05 | 0.00 | 0.05 | **36** | **TDZ 3 mg/l, NAA 0 mg/l** | 0.05 | 0.02 | 0.00 | 0.05 |
| **12** | **6-BA 3 mg/l, NAA 0.5 mg/l** | 0.40 | 0.05 | 0.00 | 0.00 | **37** | **TDZ 3 mg/l, NAA 0.5 mg/l** | 0.25 | 0.08 | 0.00 | 0.10 |
| **13** | **6-BA 3 mg/l, NAA 1.0 mg/l** | 0.25 | 0.07 | 0.00 | 0.00 | **38** | **TDZ 3 mg/l, NAA 1.0 mg/** | 0.10 | 0.04 | 0.00 | 0.00 |
| **14** | **6-BA 3 mg/l, NAA 1.5 mg/** | 0.10 | 0.03 | 0.00 | 0.00 | **39** | **TDZ 3 mg/l, NAA 1.5 mg/l** | 0.10 | 0.04 | 0.07 | 0.00 |
| **15** | **6-BA 3 mg/l, NAA 2.0 mg/l** | 0.50 | 0.03 | 0.00 | 0.00 | **40** | **TDZ 3 mg/l, NAA 2.0 mg/l** | 0.40 | 0.07 | 0.05 | 0.00 |
| **16** | **6-BA 4 mg/l, NAA 0 mg/l** | 0.74 | 0.04 | 0.05 | 0.00 | **41** | **TDZ 4 mg/l, NAA 0 mg/l** | 0.13 | 0.05 | 0.25 | 0.06 |
| **17** | **6-BA 4 mg/l, NAA 0.5 mg/l** | 0.15 | 0.04 | 0.00 | 0.05 | **42** | **TDZ 4 mg/l, NAA 0.5 mg/l** | 0.05 | 0.02 | 0.05 | 0.05 |
| **18** | **6-BA 4 mg/l, NAA 1.0 mg/l** | 0.35 | 0.06 | 0.00 | 0.00 | **43** | **TDZ 4 mg/l, NAA 1.0 mg/l** | 0.15 | 0.04 | 0.00 | 0.10 |
| **19** | **6-BA 4 mg/l, NAA 1.5 mg/** | 0.25 | 0.08 | 0.00 | 0.00 | **44** | **TDZ 4 mg/l, NAA 1.5 mg/l** | 0.20 | 0.05 | 0.10 | 0.05 |
| **20** | **6-BA 4 mg/l, NAA 2.0 mg/l** | 0.45 | 0.06 | 0.05 | 0.00 | **45** | **TDZ 4 mg/l, NAA 2.0 mg/l** | 0.30 | 0.06 | 0.25 | 0.00 |
| **21** | **6-BA 5 mg/l, NAA 0 mg/l** | 0.43 | 0.04 | 0.00 | 0.05 | **46** | **TDZ 5 mg/l, NAA 0 mg/l** | 0.30 | 0.04 | 0.20 | 0.06 |
| **22** | **6-BA 5 mg/l, NAA 0.5 mg/l** | 0.40 | 0.06 | 0.00 | 0.00 | **47** | **TDZ 5 mg/l, NAA 0.5 mg/l** | 0.20 | 0.07 | 0.00 | 0.15 |
| **23** | **6-BA 5 mg/l, NAA 1.0 mg/l** | 0.35 | 0.04 | 0.00 | 0.00 | **48** | **TDZ 5 mg/l, NAA 1.0 mg/l** | 0.15 | 0.05 | 0.00 | 0.14 |
| **24** | **6-BA 5 mg/l, NAA 1.5 mg/l** | 0.15 | 0.04 | 0.00 | 0.00 | **49** | **TDZ 5 mg/l, NAA 1.5 mg/l** | 0.15 | 0.03 | 0.00 | 0.00 |
| **25** | **6-BA 5 mg/l, NAA 2.0 mg/l** | 0.20 | 0.04 | 0.00 | 0.00 | **50** | **TDZ 5 mg/l, NAA 2.0 mg/l** | 0.25 | 0.13 | 0.00 | 0.00 |
